# Current Challenges of iPSC-Based Disease Modeling and Therapeutic Implications

**DOI:** 10.3390/cells8050403

**Published:** 2019-04-30

**Authors:** Michael Xavier Doss, Agapios Sachinidis

**Affiliations:** 1 Technology Development Division, BioMarin Pharmaceutical Inc, 105 Digital Drive, Novato, CA 94949, USA; 2 Institute of Neurophysiology and Center for Molecular Medicine, University of Cologne, Robert-Koch Str. 39, 50931 Cologne, Germany; a.sachinidis@uni-koeln.de

**Keywords:** induced pluripotent stem cells, cell replacement therapy, drug discovery, safety pharmacology, disease modeling, autologous cell therapy, allogenic cell therapy, clinical trials with stem cells

## Abstract

Induced pluripotent stem cell (iPSC)-based disease modelling and the cell replacement therapy approach have proven to be very powerful and instrumental in biomedical research and personalized regenerative medicine as evidenced in the past decade by unraveling novel pathological mechanisms of a multitude of monogenic diseases at the cellular level and the ongoing and emerging clinical trials with iPSC-derived cell products. iPSC-based disease modelling has sparked widespread enthusiasm and has presented an unprecedented opportunity in high throughput drug discovery platforms and safety pharmacology in association with three-dimensional multicellular organoids such as personalized organs-on-chips, gene/base editing, artificial intelligence and high throughput “omics” methodologies. This critical review summarizes the progress made in the past decade with the advent of iPSC discovery in biomedical applications and regenerative medicine with case examples and the current major challenges that need to be addressed to unleash the full potential of iPSCs in clinical settings and pharmacology for more effective and safer regenerative therapy.

## 1. Introduction

**Figure 1:**
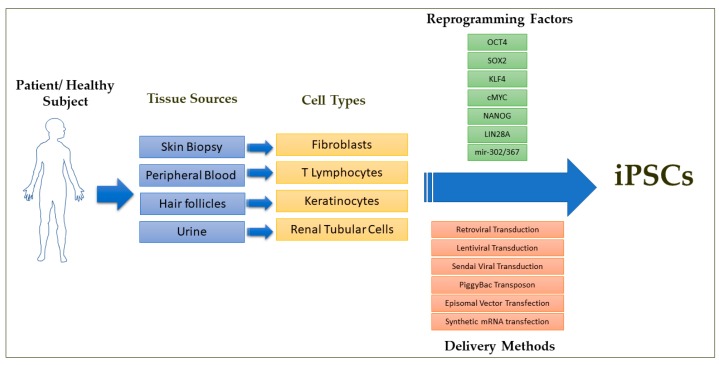
Schematic overview of iPSC derivation from a patient or healthy subject reported so far in the literature. Among the delivery methods, episomal DNA transfection and Sendai virus transduction methods are preferred for the clinical grade iPSC derivation. Although various combinations of the reprogramming factors have been used to derive iPSCs, reprogramming factor combinations free of c-Myc are preferred for the clinical applications.

One of the main distinguishing features of early mammalian development is the occurrence of a population of developmentally plastic, pluripotent stem cells that give rise to all cells that constitute the mature organism. These specialized cells can be cultured *in vitro* and are referred to as embryonic stem cells (ESCs) [1,2]. ESCs have undergone a great revolution in developmental biology through the generation of genetically engineered mice [3,4,5,6]. Since these *in vitro* grown ESCs exhibit the potential to generate all lineages of the embryo *in vivo* and can give rise to any type of somatic cells such as cardiomyocytes, smooth muscle cells, endothelial cells, neuronal cells and hepatocytes upon *in vitro* differentiation, human ESCs gained popularity as a valuable cellular source for the treatment of many degenerative diseases such as ischemic heart failure, Parkinson’s disease, Alzheimer’s disease, diabetes, spinal cord injuries and age-related macular degeneration [5,7]. Starting with an attempt to treat spinal cord injuries in 2010, there have been more than a dozen clinical trials with human ESCs to treat severe ischemic left ventricular dysfunction, age-related macular degeneration, Parkinson’s disease and diabetes, among other degenerative conditions [7,8,9]. However, the human ESC-based clinical trials suffer immensely from the ethical concerns regarding the use of cells of embryonic origin and from failed *in vitro* fertilized embryos that could result in abnormal development, and from the concerns of immune rejection after transplantation due to the allogenic origin of ESCs [10,11]. The breakthrough discovery in 2016 by Takahashi and Yamanaka enabled the reprogramming of terminally differentiated, lineage restricted adult somatic cells such as skin biopsy derived fibroblasts and peripheral blood derived T lymphocytes directly to a pluripotent state through the forced ectopic expression of the transcriptional factors OCT4, SOX2, KLF, c-MYC, NANOG and LIN28 (Figure 1) [12,13,14,15,16]. These cells, termed as induced pluripotent stem cells (iPSCs), exhibit similar gene expression, epigenetic profile and the differentiation potential to give rise to any type of somatic cells as that of ESCs.

The past 12 years following the groundbreaking discovery of iPSCs have witnessed an explosion of knowledge and enormous progress in the arena of development biology, pathophysiology and etiology for a number of diseases and disorders at the cellular level, as well as tremendous hope in the fields of cell-based regenerative medicine, high throughput drug discovery and toxicology platforms [12,17,18]. These iPSCs have been touted as a novel autologous cell source for cell replacement therapy for a number of degenerative diseases such as ischemic heart failure, Parkinson’s disease, Alzheimer’s disease, diabetes mellitus and age-related macular degeneration due to the fact that the iPSCs can be generated from any individual and that millions or even billions of clinically relevant phenotypic cells such as cardiac or neuronal cells can be derived from iPSCs without the ethical and immune rejection concerns surrounding human ESCs. Clinical trials using human iPSC-derived cellular therapeutic products have been initiated and are currently being evaluated for their efficacy and safety [9,19]. New pharmacological candidates stemming from iPSC-based high throughput screens are in the pipeline [17]. Although the iPSC field has significantly advanced, it still suffers many challenges that need to be critically addressed to transform hope into reality regarding the efficient clinical utility of these cells in regenerative medicine. This review focuses on the current challenges that pose stumbling blocks in the clinical utility of iPSC-based cell products and their applications in biomedical research, drug discovery and predictive safety pharmacology. 

## 2. iPSC-Based Disease Modeling:

The major critical component in delineating the etiology and pathophysiology of any human disease and drug discovery is the requirement of a physiologically relevant experimental model of disease, either *in vitro* or *in vivo* or both, that faithfully recapitulates the respective pathophysiology and clinical manifestations. To this end, animal modelling, most frequently with mice, has been the key player in both basic research and pharmaceutical research and development (R&D) as a non-clinical efficacy model [18]. More often, the translation of drug trials to humans from experimental animal models fails due to species differences in biological responses, leading to high failure rates as reflected by the number of new drugs approved by the US Food and Drug Administration for clinical use every year. Therefore, it is more appealing to use an appropriate human model of diseases *in vitro* that recapitulates the pathophysiological mechanisms. Human primary cell-based disease modelling would be helpful but will be limited due to insufficient expandable cellular sources from patients, in particular hard to access cells such as cardiomyocytes, neuronal cells, pancreatic beta cells and other clinically relevant cells from organs other than skin and peripheral blood. 

**Figure 2:**
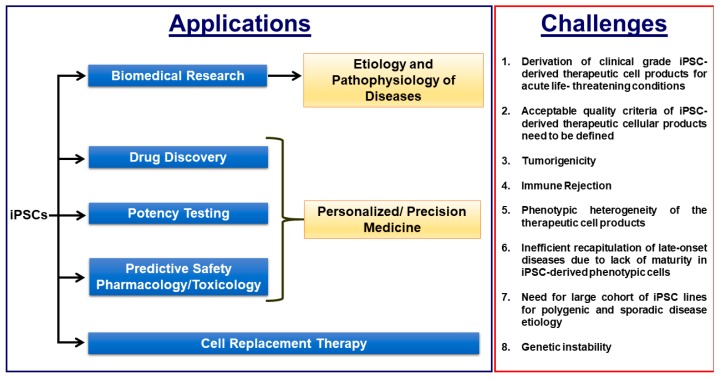
Biomedical applications of iPSCs and the critical challenges that need to be overcome for efficient clinical translation.

The so called “personalized medicine” approach in which each individual patient would receive a tailored treatment is becoming critically important in medicine, pharmacology and toxicology to overcome possible adverse side effects and to minimize the frequency of non-responders. For example, the total number of people required to take a drug in order for only one of them to benefit from its effects ranges from 5–50 for some of the highest-grossing drugs, implying the high frequency of non-responders for certain drugs [20,21]. Even more alarmingly, millions of people are hospitalized annually due to adverse side effects due to their medications, resulting in hundreds of thousands of deaths per year and highlighting the critical need for precision medicine to eliminate the adverse mortality and morbidity due to side effects of drugs [20,22]. To this end, human iPSC-based disease models (Figure 2) are a promising candidate due to their unlimited supply of clinically relevant phenotypic cells, their human origin, their derivation potential from any individual, easy accessibility and scalability and the considerable advances in understanding the etiology and progression of a diverse array of diseases such as Parkinson’s disease, Alzheimer’s disease and inherited cardiac diseases. A side-by-side comparison of human iPSC based *in vitro* and rodent *in vivo* experimental models will synergistically accelerate elucidating the pathophysiology mechanisms of diseases in basic research, novel drug discovery and safety pharmacology in a unprecedentent manner. 

Interestingly, iPSCs share a number of characteristics with cancer cells including indefinite proliferation capacity and the expression of oncogenic markers like c-MYC and metabolic signatures [23]. The generation of iPSCs from human cancer cells represents an opportunity to develop *in vitro* models of carcinogenesis for specific cancer types such as glioblastoma and gastrointestinal cancer, since the lack of a relevant model to study cancer progression has limited research which is suitable for translation to clinical settings [24,25,26,27]. Interestingly, iPSC disease models of several cancer-prone diseases such as Li–Fraumeni syndrome, Noonan syndrome, myelodysplastic syndromes, and familial adenomatous polyposis appear to be more attractive candidates in the study of cancer initiation and progression [28,29,30,31,32,33,34,35,36,37]. Also, iPSCs from somatic cells could be used to study carcinogenesis via overexpression or silencing of oncogenes and tumor suppressor genes and tracking the cellular changes and behaviors during cancer initiation and progression [38]. 

Organs-on-chips are microfluidic cell culture systems seeded with patient specific iPSC-derived phenotypic cells that serve as functional units of human organs with a controlled, dynamic condition that recapitulates the complex tissue architecture and the physio-chemical microenvironment of tissues in the human body. These systems exhibit tissue- and organ-level functions that are not recapitulated in other 2D/3D *in vitro* cell models [20]. This organ-on-chip technology is being employed to develop more physiologically relevant, cost-effective *in vitro* models for hit-to-lead and lead optimization that can more reliably predict the efficacy, toxicity and pharmacokinetics of drugs in humans [39]. These chips are increasingly used as physiologically relevant pre-clinical disease models for a wide range of different diseases such as Barth syndrome-associated cardiomyopathy, dilated cardiomyopathy, drug-induced kidney glomerular injury, blood–brain barrier function and wound healing in drug discovery and safety pharmacology platforms [20,39,40,41]. Interestingly, the patient specific organs-on-chips made up of iPSCs derived from Hutchinson–Gilford progeria syndrome patients revealed exacerbated inflammation as well as reduced vasoactive function [42,43]. 

## 3. iPSC-Based Regenerative Cell Therapy

iPSC-based regenerative therapy has been widely touted as a novel means of improving the function of degenerated organs due to ageing such as age-related macular degeneration, genetic predisposition, injury and chemotherapies. The first clinical trial to treat age-related macular generation (AMD) with autologous iPSC-derived retinal pigment epithelial cells was launched in 2014 in Japan. The AMD trial with iPSCs will be launched in the US and is expected to commence this year at NIH’s National Eye Institute (NEI). Recently, Fate Therapeutics has received approval from the Food and Drug Administration (FDA) for an iPSC trial with an off-the-shelf natural killer (NK) cell cancer immunotherapy in the US to treat solid tumors. A trial recently announced to commence in Japan aims at implanting cell sheets made of allogeneic human iPSC-derived cardiomyocytes on the epicardium of patients with heart disease [9]. These ongoing clinical trials raise tremendous hopes that the iPSC technology will bear fruit in the years to come after its Nobel Prize-winning discovery. It is noteworthy to mention that there are differences in the regulatory approval process regarding iPSC-based cell therapies across different countries. Japan has recently adopted a fast-track approval system in 2014 that allows prospective iPSC-based regenerative treatments to be commercialized as long as the treatments have been proven to be safe and the future retrospective data from the treated patients are hoped to be convincing enough for their clinical efficacy. Although Japan’s fast-track approval system on iPSC-based therapies is critically debated, more countries are expected to follow this fast-track approval path for iPSC-based regenerative cell therapies [44,45]. 

## 4. iPSC-Based Drug Discovery Platforms

Since any clinically relevant phenotypic cells such as cardiomyocytes, neuronal cells, hepatocytes, insulin secreting pancreatic beta cells and renal progenitor cells can be derived from patient-specific iPSCs in unlimited quantities for high throughput assays and iPSCs can be derived from any individual patient and healthy subjects, iPSCs have gained a lot of popularity as more reliable *in vitro* human models of diseases for accelerated drug discovery and personalized precision medicines. Large scale “-omics” analysis of diseased versus normal phenotypic cells reveal the disease-perturbed and drug-affected regulatory networks in comparison to normal ones, thereby serving as a powerful tool for drug discovery in the pharmaceutical industry [17,18,46,47,48].

## 5. Predictive Safety Pharmacology

A major bottleneck in the drug development pipeline is the predictive safety pharmacology in which the drug is evaluated for its toxicity and potency. Immortalized cell lines of cancerous origin are the most commonly used for toxicological testing. The inherent deficiencies of chromosomal and genetic aberrations due to countless passages and prolonged culture *in vitro* make them non-representative of how a normal cell behaves physiologically *in vivo.* The primary cultures of somatic cells used for toxicological screenings are heterogenous due to large batch to batch variability and hence it is very challenging to produce consistent and reproducible readouts from the toxicological screening. On the other hand, live animal models raise concerns as to the suitability for human physiology investigations for certain conditions, ethical considerations for testing certain drugs and ingredients of cosmetic products, high cost associated with animal experimentation compared to *in vitro* cultured cells and technical difficulty to automate the *in vivo* platform. Although iPSCs can also accumulate chromosomal and genetic aberrations over prolonged periods of culture similarly to immortalized cell lines, the possibility to derive iPSCs from non-cancerous tissue origin and healthy controls and the availability of more physiologically relevant phenotypic cells in millions or even in billions for high throughput drug screening makes iPSC-based disease modeling more attractive. A large cohort of iPSC-based disease models with well characterized phenotypes and diverse ethnic origins can be used for high throughput, automatable toxicological screening and potency testing platforms in a cost-effective manner to obtain more physiologically reliable readouts [18,47,49,50,51,52,53,54,55]. 

## 6. Current Challenges of iPSC-Based Disease Modelling, Cell Therapy and Drug Discovery 

### 6.1. Derivation of Clinical Grade iPSCs and Biobanking of Universal Cell Lines

The first step in generating iPSCs is the selection of the donor cell type for the reprogramming process. iPSCs have been commonly generated from dermal fibroblasts from punch-skin biopsies, T cells from peripheral blood, renal tubular cells collected from urine samples and keratinocytes isolated from plucked hair [15,16,56,57,58,59,60,61]. Several studies have reported that iPSCs retain some degree of residual epigenetic memory from the somatic cell source from which they are derived and this can lead to their biased differential potential into certain cell types depending on the donor cell source due to the incomplete resetting of the non-CpG methylation patterns during reprogramming [62,63,64,65,66]. However, it has been shown that their residual epigenetic memory diminishes as the cells are passaged in culture over a period of time [67,68]. An important concern on the choice of donor cell type is that some types of donor cells such as skin biopsy-derived dermal fibroblasts and blood cells might carry more mutational burdens and chromosomal abnormalities due to exposure to ultraviolet radiation and high cell turnover rates, especially from older donors [69,70]. The second step in the iPSC generation process is the selection of the optimal method for cellular delivery of the reprogramming factors and the optimal combination of reprogramming factors for the iPSC derivation. The earlier methods of reprogramming made use of the retroviral and lentiviral delivery methods to deliver the reprogramming factors. This raised concerns that these delivery methods would cause insertional inactivation of tumor suppressor genes and/or insertional activation of oncogenes and that the constitutive expression of the reprogramming factors would alter the iPSC characteristics and their differentiation potentials. This necessitated the use of transient, integration-free methods of delivering the reprogramming factors such as viral delivery/transient transfection methods with the use of either Sendai virus, adenovirus, episomal plasmids, minicircle plasmids, mini-intronic plasmids, PiggyBac transposons, synthetic modified mRNAs, or miRNAs [71,72,73,74,75,76,77,78]. Among these, Sendai virus, episomal DNAs and synthetic mRNAs are the commonly used approaches in basic and clinical research to derive integration-free iPSCs due to their relatively high efficiency and relative simplicity. The episomal plasmids and Sendai virus methods have been the preferred methods of choice for deriving clinical grade iPSCs (Figure 1) [70].

### 6.2. Defining the Quality Attributes of Good iPSCs and Their Differentiated Therapeutic Cellular Products

Given the huge variability across iPSC lines and their differentiated derivatives in terms of their differentiation potential, epigenetic status, tumorigenic potential, immunogenic potential, maturation characteristics, batch variability and co-occurrence of heterogenous populations of lineage subtypes and/or non-relevant cells as contaminating cell populations, the successful clinical outcome of the cell replacement therapy, in terms of efficacy and safety with these cells, largely and very critically rely on the acceptable quality criteria (Table 1) for these cells prior to the transplantation procedure in the patients. Failure to detect oncogenic mutations in these iPSCs and their derived cellular products is not a guarantor of the non-tumorigenicity aspect of the iPSC-based therapies. Even if known a priori, it may be possible that oncogenic aberrations could evade detection by the current high throughput sequencing methodologies due to sequencing errors and other technical limitations [79]. An important consideration is that the acceptable quality attributes of iPSCs and their differentiated derivatives used for clinical applications should be well defined for the safety and efficacy and this aspect is currently very poorly defined. Directed differentiation protocols and phenotypic selection based on cell surface antigens by magnetic- or flow cytometry-based sorting to yield a pure population of clinically relevant cells and strategies to improve the maturation characteristics and to gauge the magnitude of the maturation status of the cells are critically needed to improve the quality attributes of these cells for more effective cell-based regenerative therapy. A recent novel methodology making use of synthetic microRNA switches to purify the cell populations will help to improve the purity of the cell populations even if the cell surface markers are not available to tag the relevant cells [80]. The development of a consistent and reliable translational model and iPSC-derived clinical cellular products with well-defined cellular characteristics is therefore a vital pre-requisite for high throughput therapeutic applications and cell replacement therapy. Table 1 lists the minimal quality attributes required for clinical grade iPSCs and their differentiated drug products [81,82].

### 6.3. Tumorigenicity

One of the major stumbling blocks in iPSC-based cell replacement therapy is the risk of potential tumorigenicity from undifferentiated iPSCs in the cell population that will be used for cellular transplantation. The key concept is that iPSCs will almost certainly never be used in regenerative medicine if they are not able to cause a teratoma in mice [18,83,84]. iPSCs can form both teratomas and malignant tumors such as neuroblastoma and follicular carcinoma if transplanted in their undifferentiated pluripotent state *in vivo* [84]. Thus, the potential tumorigenicity risk to human patients from both teratomas and malignant tumors is quite possible if transplanted cells are contaminated with undifferentiated iPSCs. Although improved directed differentiation protocols, purification methods such as flow cytometry-/magnetic bead-based sorting and small chemical molecules that selectively cause cell death of undifferentiated iPSCs can eliminate the potential risk of tumorigenicity from potentially harmful undifferentiated iPSCs, it remains currently unknown whether these methods and tumorigenicity assays prior to transplantation are efficient and adequate enough to eliminate the risk of teratomas and malignant tumors upon transplantation in human subjects. Longer follow-up periods and very sensitive assays with the latest “-omics” approaches tailored to each and every individual patient’s genetic makeup are critically required on this end, along with analysis of cell survival, integration, durability, immune tolerance, cellular behavior, altered metabolism, functional improvements, undesired effects such as the risk of arrhythmogenicity in the case of myocardial infarction models and genetic stability in the target organs or respective tissues in the respective pre-clinical transplantation models [85]. 

### 6.4. Immune Rejection

Earlier studies in mice reported that undifferentiated iPSCs were rejected in syngeneic recipients whereas recent studies report that the differentiated cells from iPSCs do not elicit immune rejection in syngeneic settings, implying that the differentiated cells may be less immunogenic compared to undifferentiated iPSCs [86,87,88,89]. However, this could differ between cell types. For example, in humanized mice, the iPSC-derived smooth muscle cells mounted immune rejection while retinal epithelial cells did not [86,87,88,89]. Of note are the recently published results on the first clinical trial with autologous iPSC-derived retinal epithelial cells in a patient with age-related macular degeneration who showed long-term survival of transplanted cells for 25 months without immune suppression [19]. In the event that the allogenic cell therapy requires immunosuppression, there is a growing concern regarding the use of persistent immunosuppression that increases the risk of kidney failure, severe infections and tumors. The risk versus benefit ratio is highly debatable in this case. A universal immune tolerant iPSC line will be ideal to this end. Strategies to enable the allogenic iPSCs to evade both T cell- and natural killer cell-mediated immune responses have been reported [90,91]. A recently reported novel approach in which inactivation of major histocompatibility complex (MHC) class I and II genes and overexpression of CD47 in iPSCs enabled them to evade immune rejection in fully MHC mismatched allogeneic recipients and the transplanted cells survived long term without the use of immunosuppression [92]. These immune escape approaches have the potential to open the door to allogeneic iPSC-derived cell products without immune rejection concerns and complications. 

### 6.5. The Conundrum of Choosing Allogenic or Autologous iPSCs for More Efficient Cell Therapy

Despite the potential benefits of autologous iPSC therapies, there are also some limitations and challenges that need to be overcome. First, production of the clinical grade autologous iPSC -derived phenotypic cells requires a high production cost associated with individual patient-specific iPSC derivation by reprogramming along with clinical grade phenotypic cells derivation by differentiation with a stringent quality-controlled system. Second, the production of clinical grade human iPSC-derived clinically relevant phenotypic cells in a quality controlled and robust system, for example cardiomyocytes, can take approximately four months from start to finish. This can be critical since cardiac cell transplantation in chronic myocardial infarction will be less efficient than in subacute conditions [85]. More often, it may not be possible to meet the deadline for effective treatments of some disease conditions such as spinal cord injuries [6]. Therefore, the practical approach to take forward with iPSC-based cell therapy as most expert reviews conclude is the use of allogeneic iPSC-derived cell sources as opposed to autologous iPSCs with the major arguments that: (1) biobanking of a limited number of approved iPSC lines from various human leukocyte antigen (HLA)-homozygous donors that would match the majority of the population will be more efficient to provide large quantities of transplantable cells as an off-the-shelf-product from a quality controlled and rigorously tested production process; (2) regulatory clearance would be easier since any single line of such an iPSC bank could be thoroughly tested to be free of viral contamination, tumorigenicity and genome instability; and (3) the patient could more effectively benefit from the off-the-shelf product more readily in critical subacute conditions such as myocardial infarction and spinal cord injury. According to a recent estimate, it is likely to cost approximately $800,000 to produce a clinical grade autologous iPSC-derived cellular product in compliance with current good manufacturing practice (cGMP) requirements alone and approximately $10,000 to $20,000 for iPSC line generation for transplantation [93,94]. The allogenic approach can bring down the cost for iPSC-based cell therapy compared to the autologous approach and will also obviate the need for approval of individual patient-derived products by regulatory authorities [85]. On the other hand, if the transplanted cells elicit an immune response, the patients will have to be under life-long immunosuppression. Discontinuation of the immunosuppression in these patients will lead to rejection and clearance of the transplanted cells from their organs. Strategies to enable allogenic iPSC-derived therapeutic cells to evade T cell- and natural killer cell-mediated immune responses simultaneously as mentioned in the previous section would favor the use of allogenic iPSC-based cell therapy [90,91].

### 6.6. Heterogeneity of iPSC Lineage Phenotypic Cells and Cell Line Variations

One of the critical issues with iPSC-based disease modelling is the respective appropriate control. Earlier, control iPSCs were derived from family-matched, gender-matched healthy control subjects and these iPSCs exhibited a large heterogeneity and confounded the interpretation of the data due to cell line and genetic differences. The isogenic iPSC lines created by gene editing approaches from well characterized pre-existing iPSC lines from healthy control subjects can largely circumvent these cell line variation associated problems. Zinc-finger nucleases, transcription activator-like effector nucleases (TALENS), clustered regularly interspaced short palindromic repeats (CRISPR) associated (Cas) systems-9 based gene or base editing (whereby cytidine deaminase converts cytidine to uridine without the need for double strand break of DNA) enhance genetic editing efficiently in human ESCs and iPSCs [95]. This isogenic iPSC approach bridges the gap in the derivation of disease model from rare diseases with the disease-associated mutation because of its relative ease of use and high efficiency compared to other conventional tools. However, the major challenge with these gene editing methodologies is possible off-target effects. Off-target effects could be investigated with NextGen sequencing methodologies. Although it has been documented that each ESC line has its own clonal differences, iPSC lines show greater diversity than ESCs due to the residual epigenetic memory, genetic background and the characteristics acquired during reprogramming and differentiation [62,63,96]. Also, some iPSC lines exhibit characteristics of incomplete programming and reduced proliferation and differentiation potentials along with aberrant transcription and DNA methylation [97]. Evidence based criteria need to be formulated to select the completely reprogramed “bonafide” iPSC lines. 

### 6.7. iPSC Based Diseased Models for Polygenic, Sporadic and Late-Onset Diseases 

The iPSC-based disease models generated for monogenic diseases with early onset of phenotypic variations such as long QT syndrome and spinal muscular atrophy have proved to be more efficient whereas the iPSC models created for monogenic diseases with late-onset of phenotypic changes (Brugada syndrome and early repolarization syndrome), polygenic complex diseases involving association of more than one precipitating genetic mutations and interplay of various combinations of precipitating factors (schizophrenia and autism spectrum disorders), sporadic diseases for which the genetic causes have not been identified from the family history (sporadic cases of Alzheimer’s disease) and diseases that involve the interaction of more than one phenotypic cells (amyotrophic lateral sclerosis) have been more challenging. Most of these disease models failed to recapitulate the disease phenotype due to iPSC’s inherent immaturity problems (i.e., Brugada syndrome) and lack of their respective native environmental cues. On the other hand, the iPSC-based disease modeling for diseases with clear early-onset phenotypic characteristics but with unknown or undefined genetic causes proved to be very powerful in decoding the disease etiology. The approaches aimed at enhancing the maturation of iPSC phenotypes (i.e., 3D iPSC organoid models and the inclusion of a large cohort of patient-specific iPSC lines coupled with the latest gene editing methodologies and NextGen sequencing platforms) will enable us to overcome these challenges associated with the polygenic, sporadic and late-onset disease models. 

### 6.8. Need for a Large Cohort of Patient-iPSCs for More Effective Etiology and Clinical Translation

So far, the vast majority of iPSC-based disease modelling studies performed the comparative evaluation of one or a few patient-derived iPSC lines with their respective family-matched and gender-matched controls (or recently their respective isogenic cell lines created with CRISPR-Cas9 gene editing or base editing from healthy controls) and concluded the phenotypic differences observed from these cell lines to be the attributes of the respective disease mutations under study. However, such conclusions are often potentially confounded by other factors such as the genetic background and epigenetic status and the clonal variations of the iPSC lines that can dilute the observed differences during translation to the clinic in the case of complex, polygenic diseases where only disease-associated variants are known and the true causative variants are unknown. In such cases, the small number of patient-derived iPSCs will have limited potential due to large confounder effects and hence a large cohort of disease-relevant iPSC cell lines has to be used to minimize the signal-to-noise ratio. This could be challenging depending on the prevalence of the disease and the availability of patient donor cells, especially for rare diseases where the cost of iPSC derivation and the volume of lab resources required is high. To this end, the growing number of biobanks and iPSC repositories from which many iPSC lines for a particular disease are available will provide the feasibility to perform large scale studies to overcome the signal-to-noise ratio in the phenotypic differences, especially for complex polygenic diseases [70]. 

### 6.9. Lack of Maturity in iPSC-Derived Phenotypic Cells

One of the critical problems encountered in using patient-specific iPSC derived from clinically relevant phenotypic cells as *in vitro* disease models for either high throughput drug discovery, safety pharmacology or cell replacement therapy is the fact that the majority of these iPSC-derived phenotypic cells exhibit immature functional characteristics akin to their respective embryonic or fetal phenotypic cells and also contain a heterogenous mixture of varying proportions of phenotypic subtypes at the end of the differentiation protocols. For example, atrial, ventricular and nodal subtypes all occur in the same cell preparation and thereby often complicate the interpretation of experimental results. Therefore, the development of a consistent and reliable disease model with defined cellular characteristics and a homogenous population of the phenotypic cells of interest is a vital pre-requisite for high throughput therapeutic applications [98]. iPSC-based disease modelling has been widely used for early-onset diseases and proved to be successful models for early onset diseases such as long QT syndrome and spinal muscular atrophy [99,100]. However, for late-onset diseases, the disease models failed since the iPSC-derived relevant phenotypic cells lacked the adult maturation characteristics to exhibit the disease phenotype, since these cells exhibit embryonic- or fetal-like characteristics. There have been several approaches to induce the maturation of these primitive iPSC phenotypic cells such as treating the cells with mitochondrial stress inducers and inhibitors of protein degradation such as MG-132 and pyraclostrobin and ectopic overexpression of progerin, a truncated and toxic form of the lamin A protein that causes premature ageing (a condition called Hutchinson–Gilford progeria syndrome), 3D co-culture to enhance paracrine-mediated stimulation of ageing and maturation, improved formulation of cultured/conditioned medium and derivation of iPSCs from adult aged patients [101,102,103,104,105,106,107,108]. Although these approaches induced ageing of the iPSC-derived phenotypic cells to a certain extent, the maturation of these cells was only modest, since the ageing and maturation apparently seem to follow their own distinct trajectories [109,110]. An alternative approach would be the direct conversion of somatic cells such as fibroblasts into clinically relevant phenotypic cells such as neurons and cardiomyocytes to preserve the cellular ageing markers and possibly maturation state [111,112].

### 6.10. Genetic Instability

iPSCs can accumulate chromosomal abnormalities, genetic instability, copy number variants and loss of heterozygosity over a period of time in *in vitro* culture and expansion since these cells are maintained in culture for prolonged periods of time. Of particular note is that the second patient in the first clinical trial with autologous iPSCs for AMD was not treated due to the concerns regarding the genetic alterations that occurred in the patient-derived iPSCs and the iPSC-derived retinal pigment epithelial cells, implying the possible occurrence of genetic instability and aberration in the clinical grade iPSCs and their phenotypic cells [19]. It is noteworthy to highlight that exome sequencing of 140 independent human ESC lines revealed more frequent, cancer-relevant TP53 mutations and the TP53 mutant allelic fraction increased with passage number [113]. Also, an important observation is that the mesenchymal stem cells exhibit mutational burst during prolonged culture periods [114]. Therefore, adequate safety measures such as engineering the therapeutic cells with one of the suicide gene therapy approaches—either the cytosine deaminase/5-flurocytosine or the herpes simplex virus/ganciclovir—need to be devised to eliminate the chromosomally abnormal cells and cells with potential risky genetic alterations, if any, upon transplantation [18,115]. 

## 7. Conclusions and Future Perspectives

iPSCs have revolutionized personalized regenerative medicine and developmental biology within a short span of time and presented an unprecedented opportunity in deciphering the etiology and pathophysiological mechanisms of multitudes of diseases and advanced our knowledge enormously on human physiology at the cellular level. Clinical trials with iPSC-derived phenotypic cells to treat several severely debilitating degenerative diseases and organ injuries are on the way to turn hope into reality and this is just the beginning of a powerful regenerative platform with more encouraging results and optimism so far. However, iPSC-based therapeutic approaches are still in their infancy and several obstacles need to be overcome for effective translation of the true potential of iPSCs into clinical settings with uncompromised patient safety. Technological advances such as next generation sequencing and “-omics” methodologies, gene/base editing, organs-on-chips, microRNA switches, biobanking efforts to enable large cohort iPSC-based studies, automated high throughput drug discovery and toxicological platforms and the incorporation of artificial intelligence [116] in deciphering the trajectories of differentiation pathways, complex gene-regulatory networks and cellular behaviors will greatly propel iPSC-based therapeutic applications in the future. 

## Figures and Tables

**Table 1:** Minimal quality criteria required for clinical-grade iPSCs and their differentiated products.

S.No	Quality Attributes	iPSCs	iPSC-Derived Differentiated Therapeutic Product
1	Sterility and free of mycoplasma and endotoxins as required by the cGMP guidelines	✓	✓
2	Expression of pluripotency associated marks such as NANOG, OCT4, SSEA-3, SSEA-4, TRA-1-60, TRA-1-81, SOX2 [Pluritest™, hPSC Scorecard™]	✓	✕
3	Expression of differentiation markers unique to the therapeutic cellular product		✓
4	Normal Karyotype and Absence of chromosomal aberrations	✓	✓
5	Absence of undifferentiated iPSC in the final cellular drug product and free of tumorigenicity as analysed by: A. *in vivo* teratoma assay B. Whole Genome and Exome Sequencing with cancer associated gene panels C. Flow cytometry with the panel of cancer associated markers	✕	✓
6	100 % purity of the therapeutic cellular product without any contaminating other lineage cell types such as neuronal cells and hepatic cells and other cell subtypes such atrial and pacemaker cell types in therapeutic ventricular cell product, for example	✕	✓
7	Supporting *in vivo* data on the cell engraftment, durabity and functional improvement in pre-clinical models	✕	✓
8	Absence of residual reprogramming transgenes and vectors by Whole Genome and Exome Sequencing	✓	✓
9	Genotyping in case of autologous iPSCs approach [ Short Tandem Repeat Analysis]	✓	✓
10	Viability	✓	✓
